# Highly efficient lipid production in the green alga *Parachlorella kessleri*: draft genome and transcriptome endorsed by whole-cell 3D ultrastructure

**DOI:** 10.1186/s13068-016-0424-2

**Published:** 2016-01-25

**Authors:** Shuhei Ota, Kenshiro Oshima, Tomokazu Yamazaki, Sangwan Kim, Zhe Yu, Mai Yoshihara, Kohei Takeda, Tsuyoshi Takeshita, Aiko Hirata, Kateřina Bišová, Vilém Zachleder, Masahira Hattori, Shigeyuki Kawano

**Affiliations:** Department of Integrated Biosciences, Graduate School of Frontier Sciences, University of Tokyo, 5-1-5 Kashiwanoha, Kashiwa, Chiba 277-8562 Japan; Japan Science and Technology Agency (JST), CREST, 5-1-5 Kashiwanoha, Kashiwa, Chiba 277-8562 Japan; Center for Omics and Bioinformatics, Graduate School of Frontier Sciences, University of Tokyo, 5-1-5 Kashiwanoha, Kashiwa, Chiba 277-8561 Japan; Bioimaging Center, Graduate School of Frontier Sciences, University of Tokyo, 5-1-5 Kashiwanoha, Kashiwa, Chiba 277-8562 Japan; Institute of Microbiology, CAS, Centre Algatech, Laboratory of Cell Cycles of Algae, Opatovický mlýn, 379 81 Třeboň, Czech Republic; Department of Genetic Resources Technology, Faculty of Agriculture, Kyushu University, Fukuoka, Japan

**Keywords:** Genome, Green alga, Lipid body, *Parachlorella kessleri*, RNA-seq, Whole-genome sequence, Transcriptome, 3D-TEM

## Abstract

**Background:**

Algae have attracted attention as sustainable producers of lipid-containing biomass for food, animal feed, and for biofuels. *Parachlorella kessleri*, a unicellular green alga belonging to the class Trebouxiophyceae, achieves very high biomass, lipid, and starch productivity levels. However, further biotechnological exploitation has been hampered by a lack of genomic information.

**Results:**

Here, we sequenced the whole genome and transcriptome, and analyzed the behavior of *P. kessleri* NIES-2152 under lipid production-inducing conditions. The assembly includes 13,057 protein-coding genes in a 62.5-Mbp nuclear genome. Under conditions of sulfur deprivation, lipid accumulation was correlated with the transcriptomic induction of enzymes involved in sulfur metabolism, triacylglycerol (TAG) synthesis, autophagy, and remodeling of light-harvesting complexes.

**Conclusions:**

Three-dimensional transmission electron microscopy (3D-TEM) revealed extensive alterations in cellular anatomy accompanying lipid hyperaccumulation. The present 3D-TEM results, together with transcriptomic data support the finding that upregulation of TAG synthesis and autophagy are potential key mediators of the hyperaccumulation of lipids under conditions of nutrient stress.

**Electronic supplementary material:**

The online version of this article (doi:10.1186/s13068-016-0424-2) contains supplementary material, which is available to authorized users.

## Background

Microalgae accumulate oil as storage lipids (TAGs), and are ideal species for developing the highly productive strains that are essential for biofuel production [1–3]. Initial efforts to exploit microalgae for biotechnology date back to approximately 140 years ago [4]. Large-scale algal cultivation has been optimized since then, but this process was, in many cases, driven by trial and error, thus hindering the most effective utilization of algal potential [4]. Clearly, rapid development in this field can be achieved only through optimization of growth conditions guided by the results of basic research on algal physiology, morphology, and genomics.

The class Trebouxiophyceae is a major group in the green algal phylum Chlorophyta [5, 6]. One of the trebouxiophycean genera, *Chlorella*, has been well-studied with regard to its physiology and has been exploited industrially due to its high photosynthetic growth rate and excellent biomass productivity [7–9]. Recently, *Chlorella* and the closely related genus, *Parachlorella* [10], have attracted attention as potential producers of triacylglycerols (TAGs) and high value-added, long-chain fatty acid feedstocks [1, 11, 12]. Recently, a new algal species closely related to *Parachlorellakessleri*, which is also a high lipid producer, was reported from the Indian Ocean [13]. *Parachlorella kessleri*, one of three commonly described *Parachlorella* species [10, 14], is an organism that achieves very high biomass, lipid, and starch productivities [15, 16]. Importantly, *P. kessleri* is one of only a few species in which lipid productivity has been assessed not only under laboratory conditions [15–17] but also on a semi-industrial scale in outdoor photobioreactors [18], and is considered an ideal microalgal species for biofuel production.

Given their industrially verified superior growth properties, combined with high levels of lipid productivity, these species may be sustainable sources of TAG and an alternative to petroleum-based diesel fuels in the biofuel industry [1, 11]. Of the potential TAG producers, only the genome of *Nannochloropsis gaditana* [19], a member of the stramenopiles [20], has been sequenced. Moreover, a species from the same genus was shown to be capable of homologous recombination, which would thus allow for efficient gene targeting [21]. Although some green algae, particularly those belonging to the genera *Chlorella* and *Parachlorella*, are more productive lipid producers than *Nannochloropsis* [22], only few genomic sequence have been reported (*Chlorellavariabilis*, an endosymbiont of ciliates [23], and *Chlorellaprotothecoides* [24]), and a genome sequence of autotrophic *Chlorella* is currently lacking, thus limiting potential improvements in growth and productivity guided by omics techniques.

Some studies have shown that lipid content in algae can be increased by nutrient depletion [1, 4, 25–28]. Among the macronutrients (nitrogen, phosphate, and sulfur), nitrogen deprivation is widely used for stress experiments [26, 27, 29–34]. However, such stresses lower the growth rate and productivity of the system [1, 35], which is a major bottleneck for producing biofuels and byproducts on commercial scales, and therefore some studies have addressed solutions using genetic engineering [36, 37]. Sulfur deprivation is an alternative stressor for the induction of starch or TAG biosynthesis [4, 16, 38]. Notably, a high starch content was maintained for a long period under conditions of sulfur starvation, suggesting that sulfur depletion is an effective method of enhancing starch productivity [4]. In contrast to *Chlamydomonas* [39], the cellular behavior and metabolic and transcriptomic responses under sulfur-depleted culture conditions are less well-characterized in *Chlorella* species. Here, we first report the reference genome of *P. kessleri* NIES-2152. Second, we also examined the transcript patterns and cellular anatomy to evaluate responses to sulfur deprivation in the induction of TAG and carbon hydrate production [4] using phenotypic assays, RNA-seq, and three-dimensional transmission electron microscope.

## Results and discussion

The *Parachlorella* genome was sequenced and 27.8-fold pyrosequencing reads were obtained (4,365,609 reads in total). Reads were assembled into 5168 contigs and a total of 400 scaffolds were acquired. The present scaffolds and contigs are from nuclear, plastid, and mitochondria genomes. The total nuclear genome size was estimated to be 62.5 megabase pairs (Mbp) and 13,057 genes were identified (Table 1, Additional file 1: Figures S1 and S2). Thirty major scaffolds cover 48.8 % of the genome. Of the annotated genes (Additional file 1: Table S1), 49.7 % of their proteins were associated with Kyoto encyclopedia of genes and genomes (KEGG) orthology numbers.

**Table 1:** The *Parachlorella* genome assembly statistics

Characteristic	
Genome size	62.5 Mbp
GC (%)	58.30 %
Number of scaffolds	400 scaffolds
Average of scaffold size	156,382 bp
N50 scaffold size (>2k bases: 400 scaffolds)	543,086 bp
N50 scaffold size (>5k bases: 213 scaffolds)	595,262 bp
N50 scaffold size (>10k bases: 193 scaffolds)	595,262 bp
Longest scaffold size	2,165,932 bp
Number of contigs	5168 contigs
Average of contig size	11,748 bp
N50 contig size (>500 bases: 5168 contigs)	32,688 bp
N50 contig size (>5k bases: 2326 contigs)	36,671 bp
N50 contig size (>10k bases: 1643 contigs)	40,504 bp
Longest contig size	198,966 bp
Numbers of genes	13,057 genes
Average of protein length	467.0 aa
Average gene density	4.8 kb/gene
Average number of exons per gene	7.9 exons
Average exon length	176.3 bp
Average coding sequence	29.30 %

**Fig. 1:**
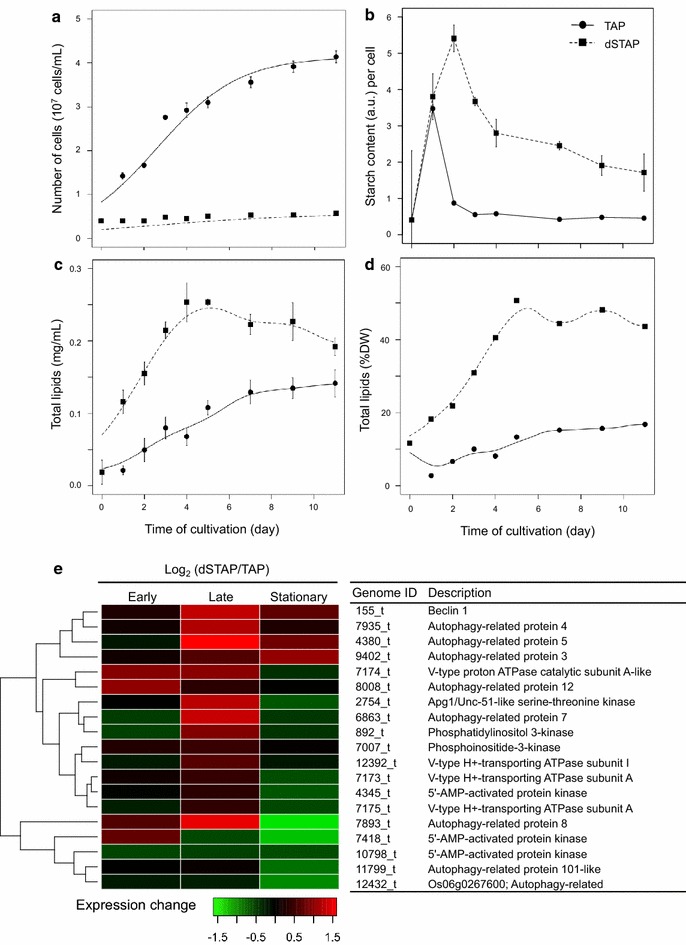
Phenotypic and transcriptomic response to sulfur deprivation. **a** Average ± SD (*n* = 3) growth in TAP (circles) and dSTAP (squares). **b** Time course of average ± SD (*n* = 3) starch accumulation. **c** Time course of average ± SD total lipid accumulation. **d** Time course of average (*n* = 3) total lipid yield (% DW). **e** Heat map of relative expression levels of autophagy-related genes. Values are indicated as the fold change (log_2_-ratio) of transcript levels under sulfur-deprived conditions relative to sulfur-replete conditions for the three culture phases (early and late log phases and stationary phase). The *dendrogram* represents hierarchical clustering based on expression values. TAP (*solid line*) and dSTAP (*dashed line*) lines are fitted curves as described in “Methods”. Real values and descriptions of autophagy-related genes are as in Additional file 7

To characterize the phenotype and transcriptome under sulfur deprivation, *P. kessleri* was batch-cultivated under continuous light (LL) and sulfur replete and deplete (±S) conditions. Growth was determined as biomass dry weight as well as cell density. As expected, growth was restricted under sulfur-depleted conditions, in contrast to its growth in tris-acetate-phosphate (TAP) medium, where a logarithmic growth phase was clearly observed (Fig. 1a). Higher starch contents per cell were observed after 2 days in culture under conditions of sulfur deprivation (6.19- and 6.62-fold changes on days 2 and 3, respectively) (Fig. 1b), consistent with results from the previous sulfur-depletion study [38].

There was a remarkable difference in total lipid content between ±S cultures. After 5 days of cultivation, *P. kessleri* growing in dSTAP (sulfur-deprived TAP) reached a yield of 0.25 mg mL^−1^ total lipids, whereas *P. kessleri* in TAP gradually accumulated total lipids over a 10-day period of batch cultivation, but achieved a yield of only 0.14 mg mL^−1^ total lipid (Fig. 1c). This finding indicates that total lipid yield was accelerated under sulfur deprivation, in contrast to cultivation in TAP medium, so that in the 5-day-old, sulfur-deprived culture, the lipid content represented up to 50.7 % of the dry weight (Fig. 1d).

A previous report showed that lipid accumulation under nitrogen-limited conditions was tightly connected with cellular processes that are related to lipogenesis, macromolecule metabolism, and autophagy in a lipid-producing yeast [40]. Here, we focus on the autophagy-related genes to show the transcriptome dynamics. During sulfur deprivation, some autophagy-related genes were upregulated in the early and late logarithmic-phase cultures (Fig. 1e). However, no genes were downregulated in the stationary-phase culture, except for *ATG3*, *ATG5*, and *beclin 1*, suggesting that autophagy and recycling of subcellular components are enhanced under sulfur-depleted conditions in the early and late logarithmic phases.

Transmission electron microscopy allows high-resolution imaging to study cellular anatomy and ultrastructure, which is inaccessible by other techniques. This is essential when studying cells in which one of the organelles is enlarged, covering other compartments and thus more or less blocking their observation by other means. Examples of such situations are cells over-producing astaxanthin [41] or lipids [42]. The main limitation of TEM is that it traditionally facilitates only two-dimensional imaging of approximately 80 nm ultrathin sections. This can be overcome by analyzing sequential sections of a single cell stacked together using three-dimensional transmission electron microscopy (3D-TEM) technology, leading to a three-dimensional image of cell ultrastructure with very high resolution. The technique was used here to study lipid hyperaccumulation within *P. kessleri* cells in the stress experiment.

**Fig. 2:**
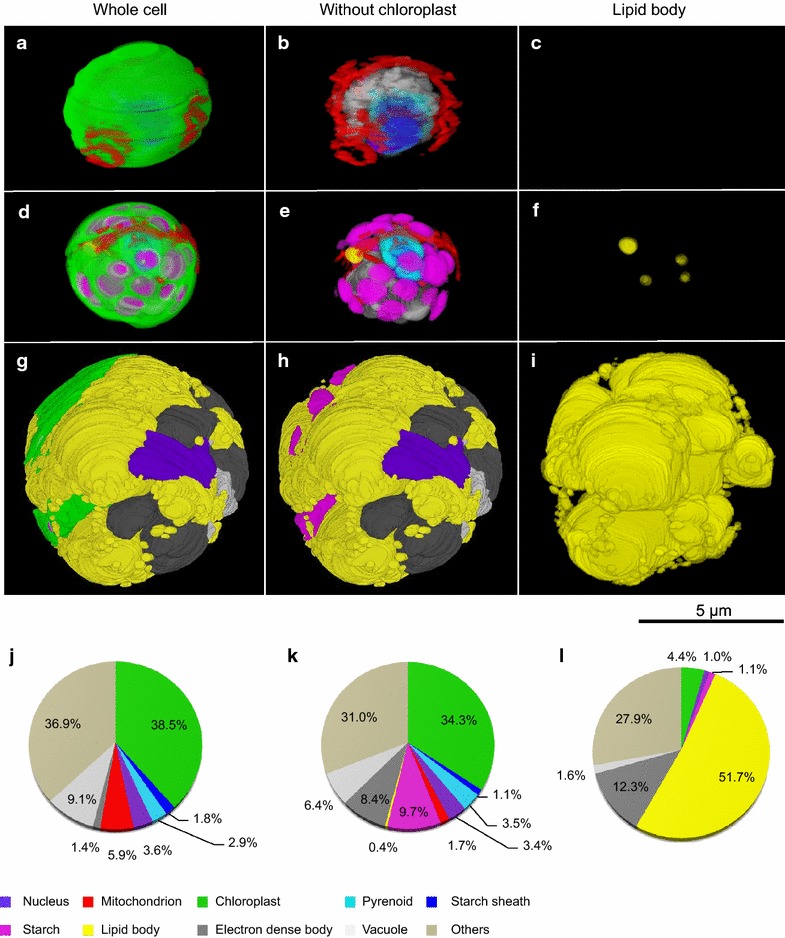
3D-TEM reconstruction and volumetric analysis of *Parachlorella kessleri.* **a**–**c** 3D-TEM image of a control cell from log-phase culture (stress-free conditions). **d**–**f** 3D-TEM image of a starch-rich phase cell (stressed conditions). **g**–**i** 3D-TEM image of a lipid-rich phase cell (stressed conditions). **j**–**l** Relative volumes of subcellular components and organelles in control cells, starch-rich, and lipid-rich cells, respectively. In each stage, 3D images are shown as a whole-cell, a whole-cell without chloroplast, and lipid body only. Volumes in the pie charts are the means of two representative cells of each stage. All subcellular and graph components are denoted by *pseudocolors* as indicated in the *color* legends. See also Additional files 2, 3, 4, and 5 as an image-rotation movie

Representative cells from three phases—logarithmic growth (control), starch-rich, and lipid-rich, were analyzed using 3D-TEM (Fig. 2, Additional files 2, 3, 4 and 5). In the logarithmic growth phase, little starch was accumulated and no lipid bodies were found (Fig. 2a–c, Additional file 2). The chloroplast and mitochondria accounted for 38.5 and 5.9 % of the relative volume, respectively (Fig. 2j). In the starch-rich phase, many starch grains were observed in the chloroplasts (Fig. 2d–f, Additional file 3), accounting for 9.7 % of the relative volume (Fig. 2k), whereas lipid bodies showed less accumulation (0.4 % of the relative volume). In this phase, the relative volume of the chloroplasts was 34.3 % (Fig. 2k).

In the lipid-rich phase, the ultrastructure and volume of the subcellular components were dramatically changed (Fig. 2g–i, Additional files 4 and 5). The most remarkable change from the starch-rich stage to the lipid-rich stage was the relative volume of chloroplasts and lipid bodies (Fig. 2i). The chloroplast was highly degenerate and its relative volume was reduced almost tenfold to 4.4 % (Fig. 2l); it was also re-located to one side of the cell periphery (Fig. 2g, Additional file 4). Concurrently, lipid bodies over-accumulated and accounted for 51.7 % of the relative volume (Fig. 2l).

Compared to the morphological changes in *Chlorella sorokiniana* under nutrient stress [42], *P. kessleri* possessed larger lipid bodies and these accounted for a considerable portion of the cell. This was accompanied by degradation of the chloroplast in the lipid-rich phase of *P. kessleri* growth (Fig. 2g, Additional files 4 and 5), but not in *C. sorokiniana*. This reflects the main differences between *P. kessleri* and other *Chlorella* species with respect to lipid production. The subcellular degradation process probably, and at least in part, involves autophagy through upregulation of ATG-related transcripts, thus enabling recycling of subcellular compartments and increased lipid accumulation.

**Fig. 3:**
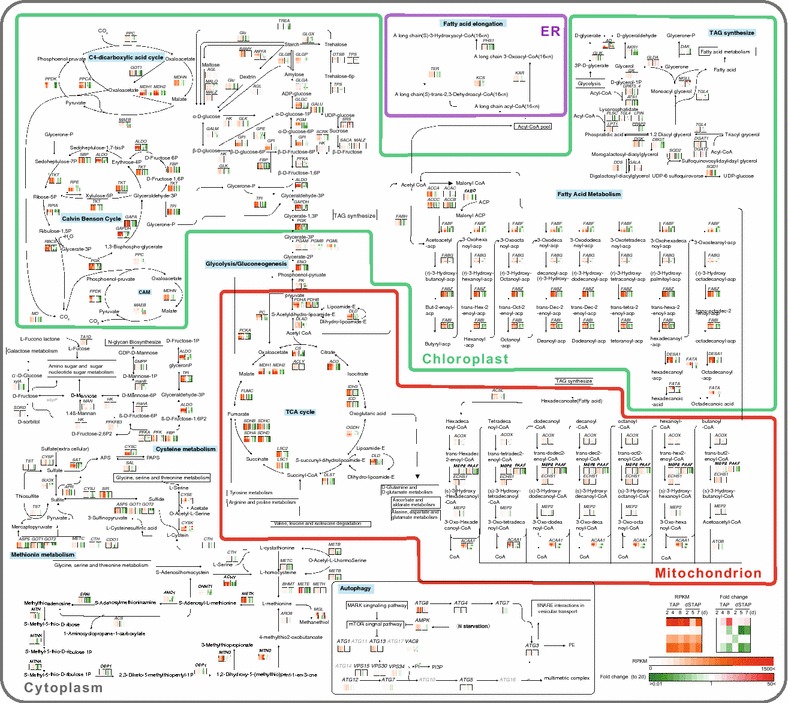
Metabolic pathway map of *Parachlorella kessleri*. The metabolic network was reconstructed based on KEGG pathway analysis. Transcriptome analysis under ±sulfur conditions is shown near the pathway as a heat map. The left heat map shows RPKM values. The right heat map shows fold changes in transcript levels relative to 2-day-old TAP culture. If the enzyme is constructed from several subunits, the transcript levels are indicated for each subunit within the heat map, as shown in a *small separate box*. Color legends are shown at the *bottom right* of the map. Gene abbreviations are as in Additional file 6

Levels of individual transcripts were expressed in a heat map and subsequently applied to a metabolic pathway map (Fig. 3, Additional file 6) and KEGG category analysis (Additional file 1: Figures S3–S5). Overall, the expression of genes involved in the Calvin–Benson, tricarboxylic acid (TCA), glyoxylate, and C4 dicarboxylic acid cycles showed decreased expression under sulfur-depleted conditions. Other metabolic pathways, such as fatty acid metabolism, autophagy, TAG, cysteine, and methanethiol synthesis were transcriptionally upregulated (Fig. 3). KEGG category analysis also indicated that cysteine and methionine metabolism (category 3: minor function category) were upregulated during the entire cultivation period under sulfur deprivation (Additional file 1: Figure S4).

Under sulfur deprivation, the sulfate transporter gene (10836_t) was highly upregulated (log_2_ value = 6.6 in the late logarithmic phase, *P* < 0.0005), together with cysteine dioxygenase (6136_t) (log_2_ value = 2.5 and 5.6 in the late logarithmic and stationary phases, respectively; *P* < 0.05 at stationary phase) (Additional file 7). This is in agreement with the induction of transcripts associated with sulfur acquisition and assimilation, synthesis of sulfur-containing amino acids, cysteine degradation, and sulfur recycling, as shown in *Chlamydomonas* [39]. In addition, the gene for *methionine*-*gamma*-*lyase* (*MGL*) was highly upregulated (Fig. 3). Degradation of l-methionine is catalyzed by MGL, resulting in methanethiol production. Because methanethiol is a sulfur-containing compound, we suspect that this metabolic pathway may be used for recycling of sulfur under sulfur-limited conditions.

It should be emphasized that transcripts responsive to sulfur deprivation also included genes for the light-harvesting complex of PSII (LHCB) (Additional file 7). Almost all genes encoding LHCBs were downregulated or did not show varying levels of expression between the ±S conditions; only one gene, *LHCB1* (1779_t), was strongly upregulated (*P* < 3 × 10^−7^). Compared to the no-stress condition, transcript levels of *LHCB1* under sulfur-depleted conditions increased by 2050- and 5106-fold in the late logarithmic and stationary phases, respectively. *LHCB1* (1779_t) is a homolog of *LHCBM9* in *Chlamydomonas* (JGI accession no: 184479). In *Chlamydomonas* [39, 43], *LHCBM9* was upregulated during sulfur deprivation. In the RNA-seq analysis [39], *LHCBM9* transcripts, which were barely detectable when cells were grown in +S medium, were upregulated by >1000-fold during sulfur deprivation, whereas transcript levels of the other *LHCBMs* declined, similarly to *P. kessleri* (Additional file 7). Because LHCBM9 contains at least three fewer sulfur-containing amino acids than other LHCBMs, it was suggested that exchange for LHCBM9 might enable recycling of sulfur-containing amino acids and their reallocation among other proteins with low sulfur amino acid contents. This process may represent an extreme measure for sulfur use that could extend cell viability under sulfur-depleted conditions. This notion was further supported by upregulation of some autophagy-related genes in the early and late logarithmic phases of sulfur-depleted cultures (Fig. 1e).

Considering transcriptionally upregulated metabolisms that involve hyperaccumulation of lipids, TAG synthesis (e.g., *DGAT1*) and autophagy emerge as candidates for the key mediator of lipid hyperaccumulation under stress conditions. The present observation based on the 3D-TEM data demonstrates that hyperaccumulation of lipids and high degradation of the chloroplast occur concurrently under stress conditions. These results illustrate that analysis based on 3D-TEM and phenotypic assays confirm the finding that the upregulation of a key mediator is involved in hyperaccumulation of lipids under nutrient stress conditions in *P. kessleri*.

Overall, this study supports the notion that basic research is required to accelerate development in the field of algal biotechnology. The genomic information on the industrially verified, biomass-producing algal species *P.kessleri* provided here will facilitate both basic and applied research not only in the field of algal-derived biofuels but will also serve as a foundation for future genetic manipulation of the TAG biosynthetic pathway (and others) in this species. Similarly, the 3D-imaging approach is a promising tool for analysis of global ultrastructural changes in whole-cells.

## Conclusions

We sequenced and analyzed the whole genome of *P. kessleri*, a high-biomass lipid-rich green alga related to *Chlorella*. Transcriptomic analysis suggested that lipid accumulation under conditions of sulfur depletion is associated not only with the induction of sulfur metabolism but also TAG synthesis, light-harvesting complexes, and autophagy. Moreover, the metabolic changes under ±sulfur conditions, autophagy-like changes in cell anatomy following lipid accumulation, were visualized by 3D-TEM ultrastructural analysis. The *Parachlorella* genome information provided by this study will facilitate basic research and further analysis of applied phycology, in addition to potential genetic manipulation, of the TAG biosynthesis pathway in this industrially verified biomass-producing species.

## Methods

### Growth conditions and DNA and RNA extraction

*Parachlorella kessleri* NIES-2152 was obtained from the National Institute for Environmental Studies in Tsukuba, Japan. For genomic DNA extraction, *P. kessleri* cells were grown at 20 ℃ under 70 µmol photons m^−2^ s^−1^ for 12 h:12 h = light (L):dark (D) cycle at 20 ℃ in TAP medium (Additional file 8: Table S2). Cells from 2-week-old cultures were harvested by centrifugation at 2500*g* for 10 min at room temperature (r.t.), and the resulting pellet (10 g of wet weight) was immediately frozen with liquid nitrogen. DNA extraction was performed using the DNeasy Plant Maxi Kit (Qiagen, Hilden, Germany) according to the manufacturer’s protocol. For RNA extraction, cells were grown in TAP or dSTAP (Additional file 8: Table S3) medium in a 500 ml flask (Iwaki, Tokyo, Japan) under 100 µmol photons m^−2^ s^−1^ for 12 h:12 h = light (L):dark (D) cycle at 20 ℃. Cells from 2-week-old cultures were harvested by centrifugation at 2500*g* for 10 min at r.t., and the resulting pellet was immediately frozen with liquid nitrogen. Total RNA extraction was performed using the Sepasol^®^-RNA I Super G kit (Nacalai Tesque, Kyoto, Japan) followed by poly (A) mRNA purification with Dynabeads Oligo (dT) (Life Technologies, Carlsbad, USA) in cultures grown under conditions of ±sulfur in the early (2 days old) and late (4–5 days old) logarithmic phases and stationary phase (7–8 days old). In this experiment, 12.5 mL of the Sepasol^®^-RNA I Super G for every 0.5 g of cells were used in each experimental group. The purification was performed in three rounds. In the first round, 150 µg of total RNA were used for mRNA purification using 1 mg of Dynabeads Olig (dT) according to the manufacturer’s protocol with modifications. Finally, 500 ng of mRNA were obtained.

### Genomic and mRNA sequences and annotation

The genomic sequence of *P. kessleri* NIES-2152 was determined using 454 pyrosequencing for single-end (SE) and paired-end (PE, 8 kb-span library) data. We generated 3,561,169 reads by SE and 804,440 reads by PE, which provided 27.8-fold coverage of the genome. The assembly of the obtained sequence data resulted in the generation of 400 scaffolds using Newbler version 2.8 (Roche, Branford, CT, USA). The total scaffold length was 2,653,566 bp with a G + C content of 58.4 %. Protein-encoding regions were predicted using the GeneMark-ES [44]. We annotated 13,057 predicted genes using the BLASTP program [45] (e-value cut-off of 1E−5) with the NCBI-nr and KEGG databases.

The mRNA sequencing libraries of *P. kessleri* NIES-2152 were constructed using the ion total RNA-Seq Kit v2 (Life Technologies, Carlsbad, USA) and the libraries were sequenced using an Ion PGM sequencer (Life Technologies, Carlsbad, USA). We generated 4,220,822 reads (2d: 2-day-old culture in TAP), 5,681,304 reads (4d: 4-day-old culture in TAP), 4,742,155 reads (8d: 8-day-old culture in TAP), 4,144,745 reads (ds_2d: 2-day-old culture in dSTAP), 4,220,451 reads (ds_5d: 5-day-old culture in dSTAP), and 3,694,851 reads (ds_7d: 7-day-old culture in dSTAP) from six mRNA samples. The *Parachlorella* gene IDs and full descriptions of protein names are given in Additional files 6 and 7.

### Reconstruction of KEGG pathway map and mRNA expression analysis

The RNA-seq reads were mapped to the predicted genes using Newbler v.2.8. Comparative expression analyses were performed using standardized reads per kilobase of exon per million mapped sequence reads (RPKM) values. Functional annotation descriptions were assigned by BLASTP [45] with the KEGG database (e-value cut-off of 1E−10). The metabolic network was reconstructed using KEGG mapper (http://www.genome.jp/kegg/) with the KO numbers as objects shown in Additional file 6. The resulting KEGG map was redrawn manually using Adobe Illustrator v. 16.0.4 (Adobe Systems). An abbreviated gene names are given in Additional file 6. A heat map of the autophagy-related transcriptomes was generated using the *heatmap2* function from the *Gplots* package v. 2.16.0 in the R statistical software v. 3.1.0 (http://www.R-project.org/). Statistical testing for gene expression was performed in R with DESeq [46] using the no replicate method. The *P* values are provided in Additional file 9. We performed KEGG category analysis in which different sequences were treated as different genes.

### Phenotypic assays for biomass and lipid production

Pre-cultures were grown under 100 µmol photons m^−2^ s^−1^ for 12 h:12 h = L:D cycle at 23 ℃. Cells of 4-day-old cultures were centrifuged at 1500*g* for 5 min at r.t. and collected as a pellet. The pellet was re-suspended and inoculated into 500 mL of TAP or dSTAP medium (the initial concentration of the culture was ~7 × 10^6^ cells mL^−1^). The batch cultures were grown under continuous illumination (100 µmol photons m^−2^ s^−1^) at 21–23 °C and agitated with a magnetic stirrer (MGM-66, Shibata, Tokyo, Japan) at 100–150 rpm.

Cells were counted using a particle counter (CDA-1000, Sysmex, Kobe, Japan). A general linear model was fit to values of the cell numbers in each condition for growth curves. For dry weight determination, an aliquot of cell culture was sampled into a pre-weighed sampling tube and centrifuged at 6000*g* for 5 min at r.t. The supernatant was then removed and the cell pellet was dried for at least 3 h to a constant weight at 105 ℃. The sampling tube was weighed using a precision analytical balance (NewClassic MS, Mettler Toledo, MD). For total lipid extraction and measurements, we used a previously described method [16]. Briefly, total lipids were extracted using methyl-*tert*-butyl ether (MTBE) [47], and the weight of total lipids was measured gravimetrically using a precision analytical balance. Starch content was quantified using the Lugol staining method as described [48]. Growth curves were estimated using a general linear model (*glm* function), and other curves (except for starch assay) were fitted using the *ksmooth* function in R statistical software v. 3.1.0.

## 3D-TEM analysis

We observed three representative stages: control (4 days old in TAP under LD), starch-rich [6 days old in dSTAP under light/dark cycle (LD)], and lipid-rich (6 days old in dSTAP under LL). Cells at each stage were pre-fixed for 2 h with 2.5 % glutaraldehyde, post-fixed with 1 % OsO_4_ for 2 h at r.t., and then rinsed with 0.05 M sodium cacodylate buffer (pH 7.2). The fixed cells were then dehydrated using a graded ethanol series, incubated in ethanol:acetone = 1:1 and finally suspended in 100 % acetone at r.t. The dehydrated samples were infiltrated with increasing concentrations of Spurr’s resin [49] in acetone and finally with 100 % Spurr’s resin. Ultrathin serial sections were cut on a Reichert Ultracut S ultra-microtome (Leica, Vienna, Austria) using a diamond knife. Serial sections were mounted on copper grids coated with polyvinyl formvar films and stained in 3 % aqueous uranyl acetate and lead citrate [50]. The sections were observed at 100 kV using an H-7650 transmission electron microscope (Hitachi High Technologies, Tokyo, Japan).

3D-TEM imaging followed a previously described method [41]. Briefly, contours of each subcellular element (e.g., nucleus, chloroplast, lipid body) were traced manually. After binarization of the traced subcellular elements, 3D images were reconstructed using TRI/3D SRFIII software (Ratoc System Engineering, Tokyo, Japan). Voxel-based volumetric analyses were performed using the TRI/3D SRFIII system and data were presented as the means of two representative cells.

### Nucleotide sequence accession numbers

The *P. kessleri* NIES-2152 whole genome has been deposited in DDBJ/EMBL/GenBank under the accession BBXU01000001-BBXU01003651 (BioProject number: PRJDB3487).

## Additional files

10.1186/s13068-016-0424-2 Number of genes versus size (nt in length) of genes. **Figure S2.** Number of genes versus number of exons. **Figure S3.** KEGG category analysis in energy metabolism. **Figure S4.** KEGG category analysis in amino acid metabolism. **Figure S5.** KEGG category analysis in lipid metabolism. **Table S1.** Percentage of genes annotated.
10.1186/s13068-016-0424-2 3D-reconstruction movie (mov format) of a control cell without a chloroplast. The color legends used in the movie are the same as in Fig. 2.
10.1186/s13068-016-0424-2 3D-reconstruction movie (mov format) of a starch-rich cell without a chloroplast. The color legends used in the movie are the same as in Fig. 2.
10.1186/s13068-016-0424-2 3D-reconstruction movie (mov format) of a lipid-rich cell. The color legends used in the movie are the same as in Fig. 2.
10.1186/s13068-016-0424-2 3D-reconstruction movie (mov format) of lipid bodies in a lipid-rich cell.
10.1186/s13068-016-0424-2 Gene ID, KO numbers, and full description of gene names corresponding to the metabolic pathway map.
10.1186/s13068-016-0424-2 RPKM and fold change of all *Parachlorella* transcripts.
10.1186/s13068-016-0424-2 Recipe of TAP medium. **Table S3.** Recipe of dSTAP medium.
10.1186/s13068-016-0424-2 *P* values of the present RNA-seq analysis.

## Abbreviations

dSTAP: sulfur-deprived TAP
KEGG: Kyoto encyclopedia of genes and genomes
LD: light/dark cycle
LL: continuous light
NIES: National Institute for Environmental Studies, Tsukuba, Japan
RPKM: reads per kilobase of exon per million mapped sequence reads
TAG: triacylglycerol
TAP: tris-acetate-phosphate
TCA: tricarboxylic acid
3D-TEM: three-dimensional transmission electron microscopy
