# Supplementary material for: Highly efficient lipid production in the green alga Parachlorella kessleri: draft genome and transcriptome endorsed by whole-cell 3D ultrastructure
Source: Biotechnol Biofuels. 2016 Jan 25;9:13. doi: 10.1186/s13068-016-0424-2 (PMC4724957; doi:10.1186/s13068-016-0424-2)
Supplement: Supplementary file 1 — 10.1186/s13068-016-0424-2 Number of genes versus size (nt in length) of genes. Figure S2. Number of genes versus number of exons. Figure S3. KEGG category analysis in energy metabolism. Figure S4. KEGG category analysis in amino acid metabolism. Figure S5. KEGG category analysis in lipid metabolism. Table S1. Percentage of genes annotated. [file 13068_2016_424_MOESM1_ESM.pdf]

Additional file 1: Figures and Table

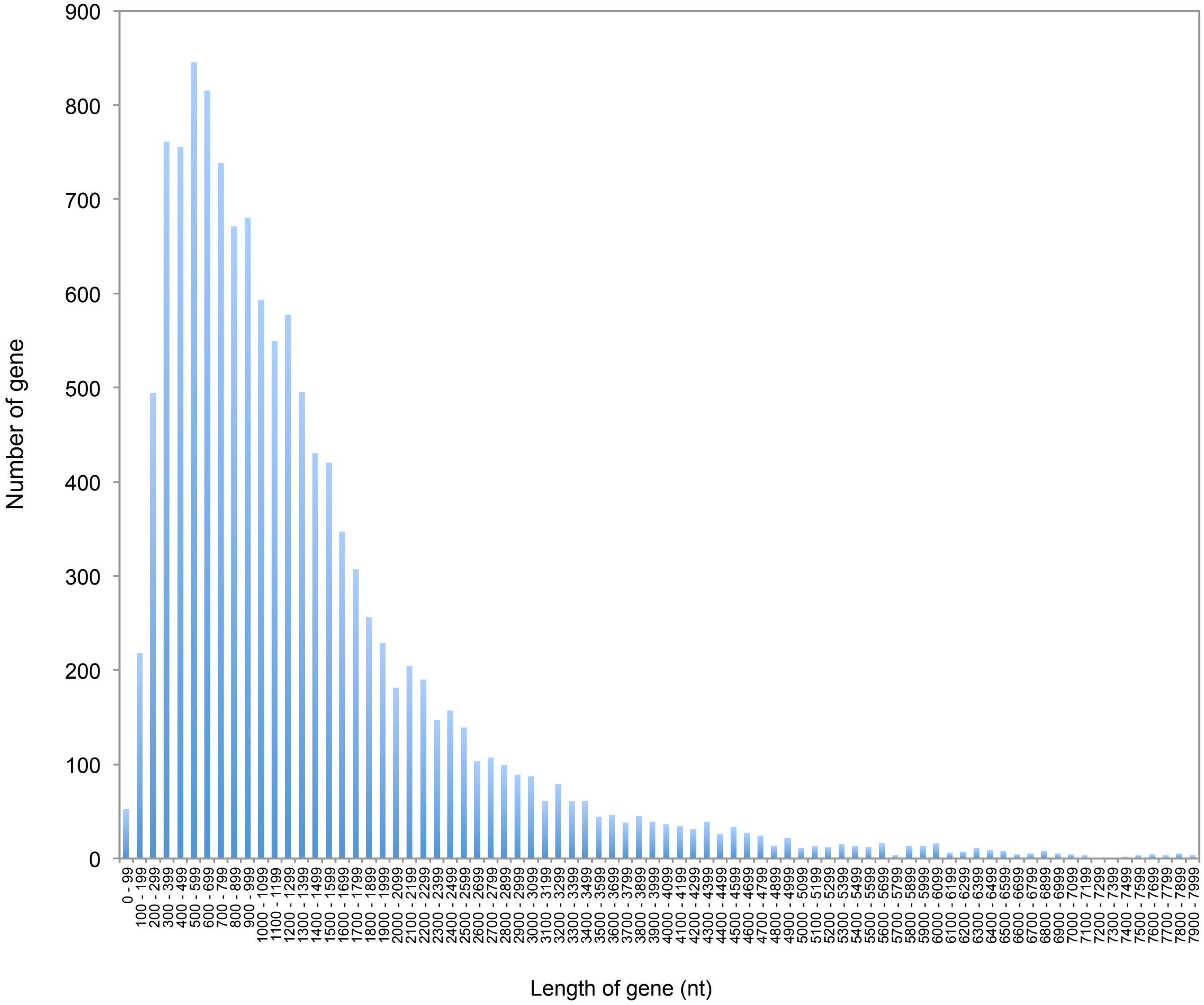

Figure S1. Number of genes versus size (nt in length) of genes.

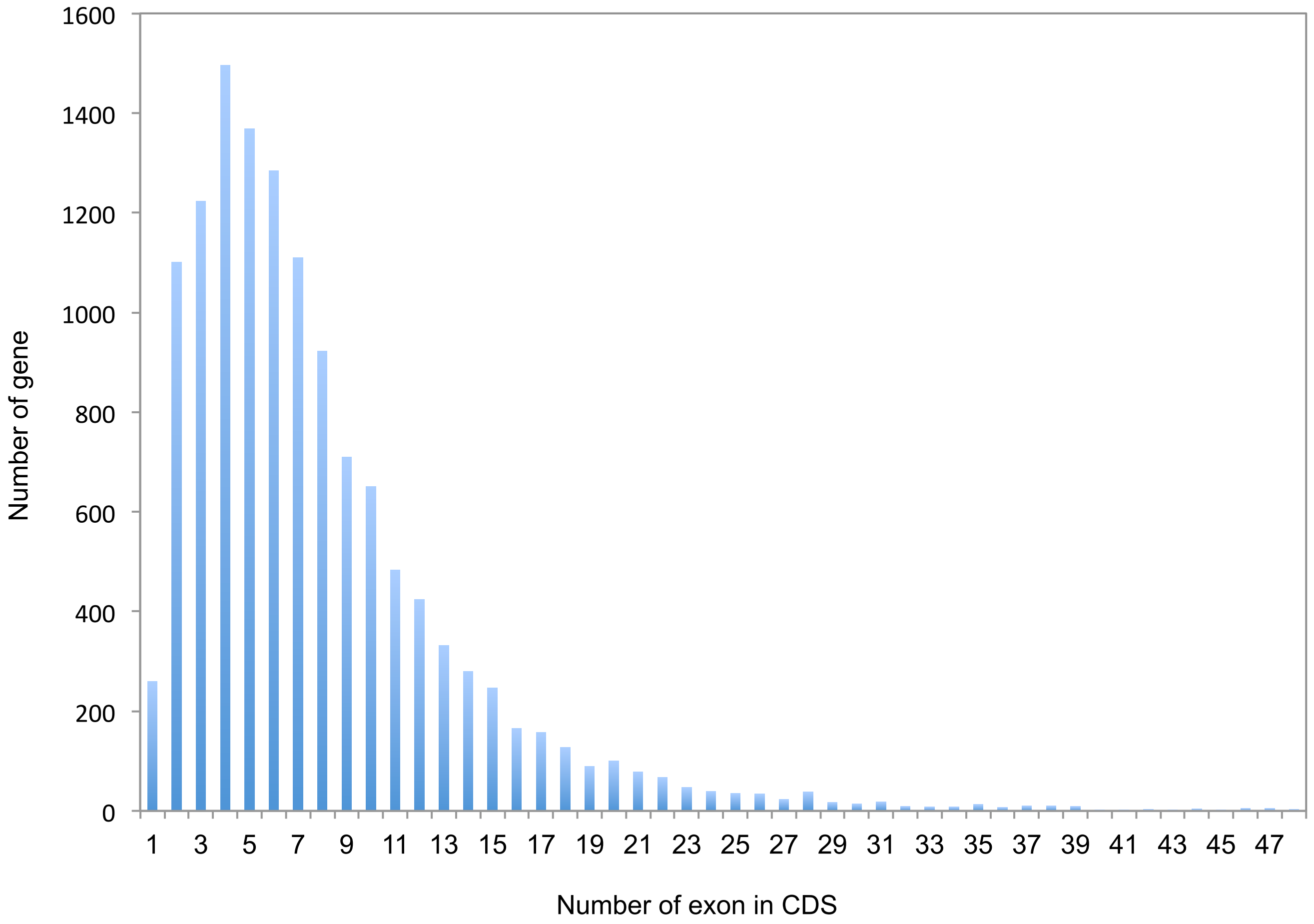

Figure. S2. Number of genes versus number of exons.

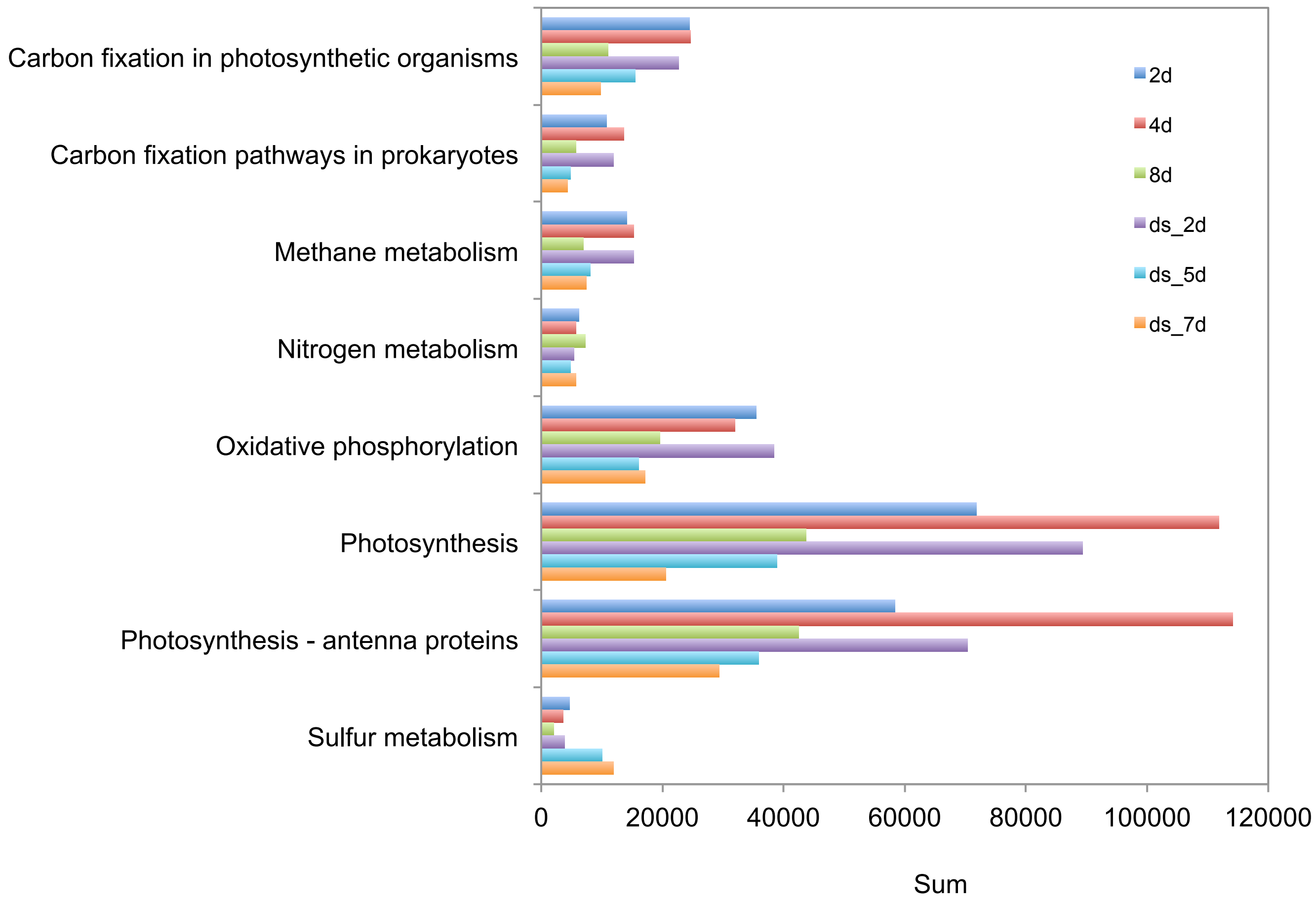

Figure. S3. KEGG category analysis in energy metabolism. 2d: 2-day-old culture in TAP, 4d: 4-day-old culture in TAP, 8d: 8-day-old culture in TAP, ds_2d: 2-day-old culture in dSTAP, ds_5d: 5-day-old culture in dSTAP, ds_7d: 7-day-old culture in dSTAP.

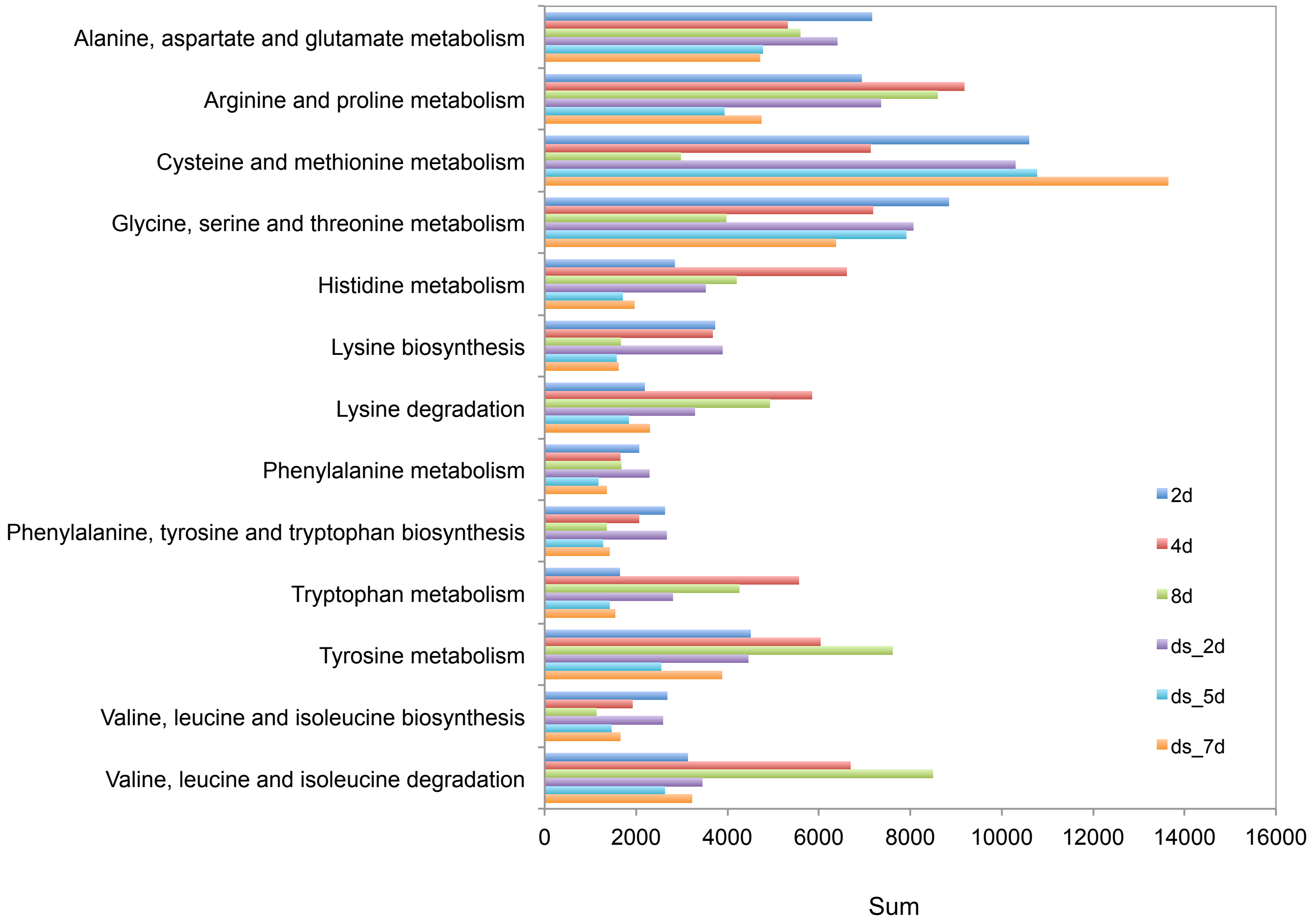

Figure. S4. KEGG category analysis in amino acid metabolism. 2d: 2-day-old culture in TAP, 4d: 4-day-old culture in TAP, 8d: 8-day-old culture in TAP, ds_2d: 2-day-old culture in dSTAP, ds_5d: 5-day-old culture in dSTAP, ds_7d: 7-day-old culture in dSTAP.

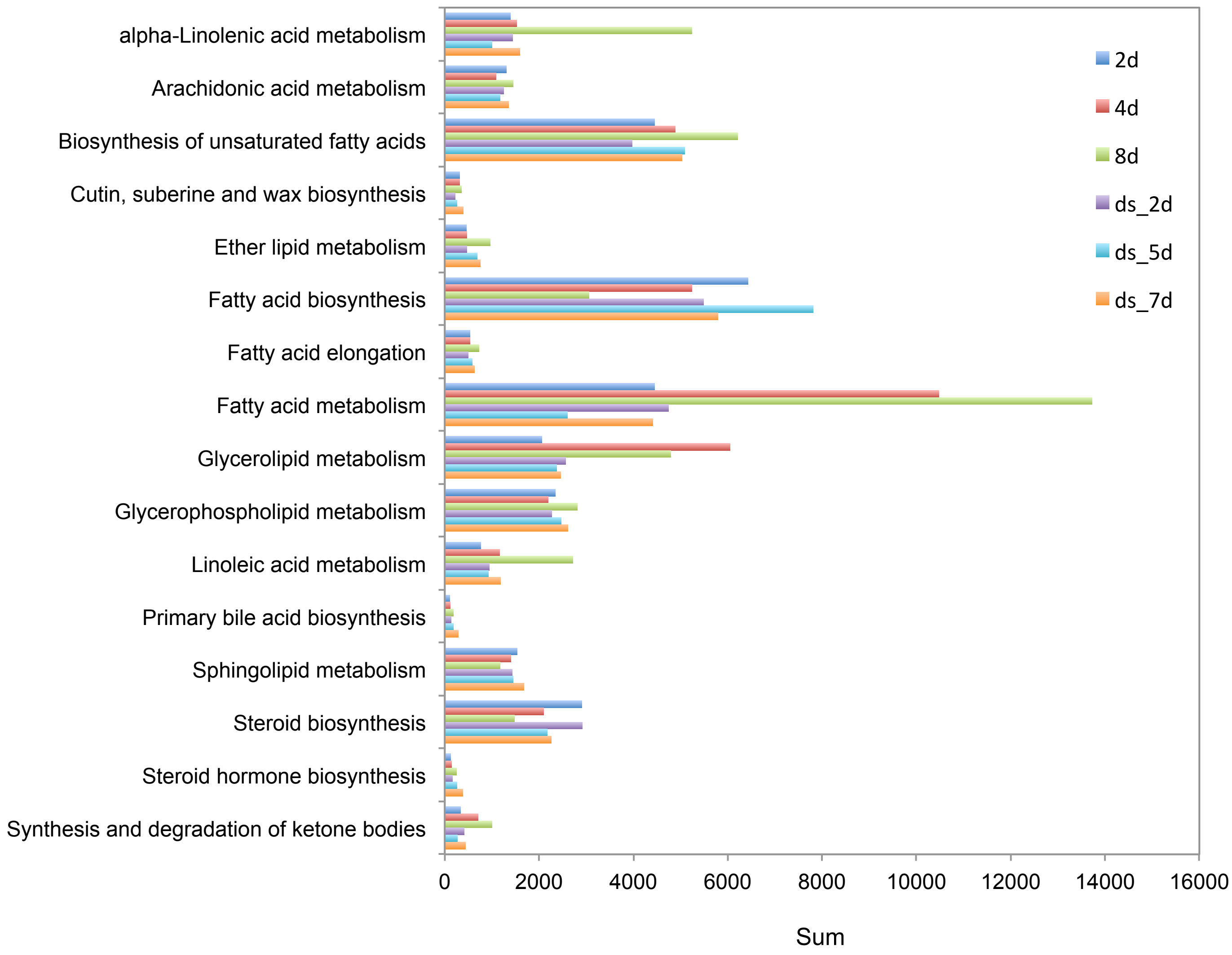

Figure S5. KEGG category analysis in lipid metabolism. 2d: 2-day-old culture in TAP, 4d: 4-day-old culture in TAP, 8d: 8-day-old culture in TAP, ds_2d: 2-day-old culture in dSTAP, ds_5d: 5-day-old culture in dSTAP, ds_7d: 7-day-old culture in dSTAP.

Table S1: Percentage of genes annotated.

KEGG category	% of genes annotated
Amino acid metabolism	4.3%
Biosynthesis of other secondary metabolites	0.6%
Cancers	2.9%
Carbohydrate metabolism	6.1%
Cardiovascular diseases	0.2%
Cell communication	0.5%
Cell growth and death	2.4%
Cell motility	0.2%
Circulatory system	0.2%
Development	0.3%
Digestive system	1.1%
Endocrine and metabolic diseases	0.2%
Endocrine system	1.2%
Energy metabolism	3.5%
Environmental adaptation	0.8%
Excretory system	0.5%
Folding, sorting and degradation	3.6%
Glycan biosynthesis and metabolism	1.2%
Immune diseases	0.8%
Immune system	1.4%
Infectious diseases	4.6%
Lipid metabolism	3.5%
Membrane transport	0.7%
Metabolism of cofactors and vitamins	2.3%
Metabolism of other amino acids	1.1%
Metabolism of terpenoids and polyketides	0.8%
Nervous system	1.5%
Neurodegenerative diseases	1.9%
Hypothetical protein	18.1%
Nucleotide metabolism	2.5%
Replication and repair	2.4%
Sensory system	0.3%
Signal transduction	3.7%
Signaling molecules and interaction	0.1%
Substance dependence	0.8%
Transcription	2.0%
Translation	4.2%
Transport and catabolism	2.3%
Xenobiotics biodegradation and metabolism	2.3%
Gene of non-assigned KEGG function	12.8%
