# Supplementary material for: Highly efficient lipid production in the green alga Parachlorella kessleri: draft genome and transcriptome endorsed by whole-cell 3D ultrastructure
Source: Biotechnol Biofuels. 2016 Jan 25;9:13. doi: 10.1186/s13068-016-0424-2 (PMC4724957; doi:10.1186/s13068-016-0424-2)
Supplement: Supplementary file 8 — 10.1186/s13068-016-0424-2 Recipe of TAP medium. Table S3. Recipe of dSTAP medium. [file 13068_2016_424_MOESM8_ESM.pdf]

Table S2. Recipe of TAP medium

Component	Stock Solution (mg/mL)	Quantity Used*
NH_4Cl	200	2.0 mL
$CaCl_2 \cdot 2H_2O$	100	0.5 mL
$MgSO_4 \cdot 7H_2O$	300	1.0 mL
K_2HPO_4	100	1.0 mL
KH_2PO_4	100	1.0 mL
Hutner's trace elements	See following recipe	1.0 mL
Acetic acid	-	1.0 mL
Tris (hydroxymethyl) aminomethane	-	2.42 g

*To prepare: make up to 1 liter with distilled water (DW). Autoclave at 121°C for 20 minutes.

Recipe of Hutner's trace elements

Component	Quantity Used (per 100 mL DW)**
$Na_2EDTA \cdot 2H_2O$	5.000 g
$ZnSO_4 \cdot 7H_2O$	2.200 g
H_3BO_3	1.140 g
$MnCl_2 \cdot 4H_2O$	0.506 g
$FeSO_4 \cdot 7H_2O$	0.499 g
$CoCl_2 \cdot 6H_2O$	0.161 g
$CuSO_4 \cdot 5H_2O$	0.157 g
$(NH_4)_6Mo_7O_{24} \cdot 4H_2O$	0.110 g
KOH	~1.6 g

**Dissolve each of the above components, and then bring the final volume up to 100 mL with DW.

Table S3. Recipe of dSTAP medium

Component	Stock Solution (mg/mL)	Quantity Used*
NH_4Cl	200	2.0 mL
$CaCl_2 \cdot 2H_2O$	100	0.5 mL
$MgCl_2 \cdot 6H_2O$	254	1.0 mL
K_2HPO_4	100	1.0 mL
KH_2PO_4	100	1.0 mL
Hutner's trace elements for dSTAP	See following recipe	1.0 mL
Acetic acid	-	1.0 mL
Tris (hydroxymethyl) aminomethane	-	2.42 g

*To prepare: make up to 1 liter with DW. Autoclave at 121°C for 20 minutes.

Recipe of Hutner's trace elements for dSTAP

Component	Quantity Used (per 100 mL DW)**
$Na_2EDTA \cdot 2H_2O$	5.000 g
$ZnCl_2$	1.040 g
H_3BO_3	1.140 g
$MnCl_2 \cdot 4H_2O$	0.506 g
$FeCl_3 \cdot 7H_2O$	0.485 g
$CoCl_2 \cdot 6H_2O$	0.161 g
$CuCl_2 \cdot 2H_2O$	0.107 g
$(NH_4)_6Mo_7O_{24} \cdot 4H_2O$	0.110 g
KOH	~1.6 g

**Dissolve each of the above components, and then bring the final volume up to 100 mL with DW.
